# Supplementary material for: Evidence supported by Mendelian randomization: impact on inflammatory factors in knee osteoarthritis
Source: Front Med (Lausanne). 2024 May 28;11:1382836. doi: 10.3389/fmed.2024.1382836 (PMC11165061; doi:10.3389/fmed.2024.1382836)
Supplement: Supplementary file 1 [file Data_Sheet_1.ZIP › data/GCST90274801_5e6/801.leaveoneout.pdf]

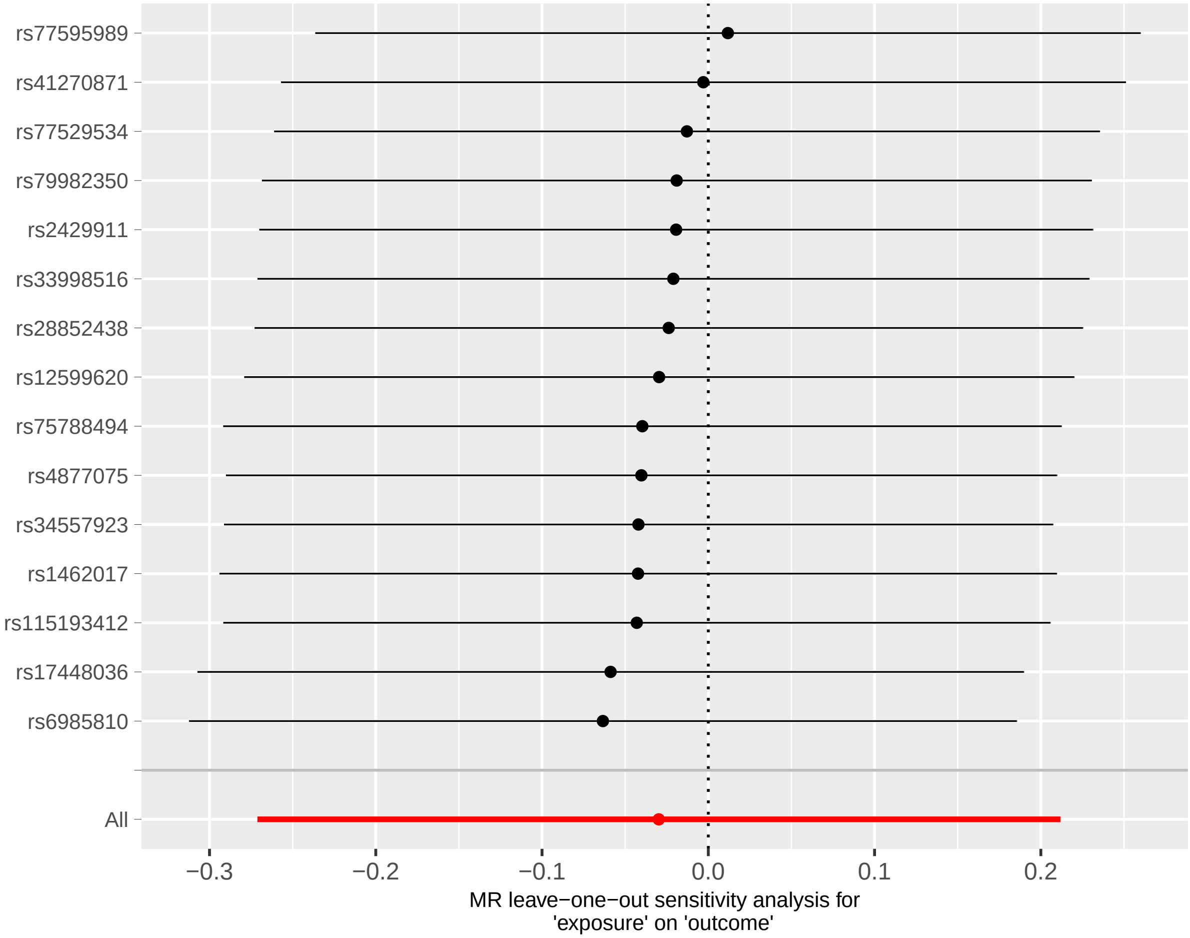
rs77595989
rs41270871
rs77529534
rs79982350
rs2429911
rs33998516
rs28852438
rs12599620
rs75788494
rs4877075
rs34557923
rs1462017
rs115193412
rs17448036
rs6985810
All
−0.3
−0.2
−0.1
0.0
0.1
0.2
MR leave−one−out sensitivity analysis for
'exposure' on 'outcome'
